# Tensile Deformation Mechanism of an *In Situ* Formed Ti-Based Bulk Metallic Glass Composites

**DOI:** 10.3390/ma17184486

**Published:** 2024-09-12

**Authors:** Haiyun Wang, Na Chen, Huanwu Cheng, Yangwei Wang, Denghui Zhao

**Affiliations:** 1 School of Materials Science and Engineering, Beijing Institute of Technology, Beijing 100081, China; wanghaiyunsophia@yeah.net (H.W.);; 2 China Ordnance Industrial Standardization Research Institute, Beijing 100089, China

**Keywords:** bulk metallic glass composites, tension deformation, serration, local amorphization, β to α transition

## Abstract

Ti-based bulk metallic glass composites (BMGMCs) containing an *in situ* formed metastable β phase normally exhibit enhanced plasticity attributed to induced phase transformation or twinning. However, the underlying deformation micromechanism remains controversial. This study investigates a novel deformation mechanism of Ti-based BMGMCs with a composition of Ti_42.3_Zr_28_Cu_8.3_Nb_4.7_Ni_1.7_Be_15_ (at%). The microstructures after tension were analyzed using advanced electron microscopy. The dendrites were homogeneously distributed in the glassy matrix with a volume fraction of 55 ± 2% and a size of 1~5 μm. The BMGMCs deformed in a serrated manner with a fracture strength (σ_f_) of ~1710 MPa and a fracture strain of ~7.1%, accompanying strain hardening. The plastic deformation beyond yielding was achieved by a synergistic action, which includes shear banding, localized amorphization and a localized BCC (β-Ti) to HCP (α-Ti) structural transition. The localized amorphization was caused by high local strain rates during shear band extension from the amorphous matrix to the crystalline reinforcements. The localized structural transition from BCC to HCP resulted from accumulating concentrated stress during deformation. The synergistic action enriches our understanding of the deformation mechanism of Ti-based BMGMCs and also sheds light on material design and performance improvement.

## 1. Introduction

Bulk metallic glasses (BMGs), as a prospective structural material, have attracted substantial attention due to their outstanding mechanical, chemical and physical properties [1,2,3]. Nevertheless, strain softening behaviour and brittleness at ambient temperatures have limited their applications as structural materials [4,5,6]. To tackle this issue, numerous strategies have been proposed in recent years, and metallic glass matrix composites (MGMCs) have proved to be an effective solution [7,8]. In efforts to enhance the tensile ductility of BMGs, bulk metallic glass composites (BMGCs) with *in situ* precipitated reinforcements during solidification have been developed [9]. CuZr-based BMGCs with B2 CuZr precipitates [10,11] and Zr/Ti-based BMGCs containing β-Zr/Ti dendrites [9,12,13,14] are the two most extensively studied groups of BMGCs. CuZr-based BMGMCs exhibit excellent room-temperature plastic performance in the plastic deformation as a result of the martensitic phase transformation of metastable B2 CuZr phase during plastic straining [11].

Ti-based BMGMCs containing β-phase are widely investigated due to their large forming sizes and repeatable microstructures. Their mechanical properties can be tuned via adjusting the volume fraction, the size and the stability of the β-phase [15,16]. The reported deformation mechanisms include the formation of dense dislocation walls in the dendrites [17], dislocation mediation [14,18], the twinning of the β-phase [19], a transition from β to α (hexagonal, space group of P63/mmc) [20], a transition from β to ω (hexagonal, space group of P6/mmm) [21,22,23] and a reversible martensitic transition from β to α [24,25]. The occurrence of phase transformation necessitates an appropriate metastability of the *in situ* formed β phase. If it is excessively stable, no phase transformation will occur during deformation. Conversely, if it is overly unstable, decomposition will take place during solidification [14,21]. As reported, the HCP structure could be transformed from the BCC structure through two modes [26]. The first mode involves atomic shuffling displacement, while the second one entails compression/dilation of the (1 1 0)_BCC_ planes along the [0 0 1] or [1 1 0] directions.

Traditional Ti-based BMGMCs, on the other hand, often demonstrate strain softening. This is attributed to the insufficient compensation of shear softening in the amorphous matrix by strain hardening generated through dislocation entanglement in the crystalline reinforcements. In addition, there are no serration characteristics because the propagation of the shear band is well restricted within the intercrystalline amorphous region. Recent research has shown that deformation-induced phase transformation or twinning can overcome the strain softening drawback in some novel Ti-based BMGMCs [27,28,29]. The underlying deformation micromechanism of Ti-based BMGMCs remains elusive if they exhibit serrated deformation behavior without strain softening.

In this study, we present a novel plastic deformation mechanism under the tensile deformation of a Ti-based BMGMC containing metastable β-phase dendrites. The BMGMCs undergo a macroscopic serrated flow without obvious work hardening or work softening beyond yielding. The plastic deformation is achieved by a synergistic action including shear banding, localized amorphization and a localized BCC to HCP structural transition.

## 2. Materials and Methods

The bulk samples with a composition of Ti_42.3_Zr_28_Cu_8.3_Nb_4.7_Ni_1.7_Be_15_ (at%) were provided by Institute of Metal Research, Chinese Academy of Sciences. The master ingot was fabricated by a vacuum arc melting method. It was produced by arc melting a mixture of elemental metals with high purity of up to 99.99% in a Ti-gettered Argon atmosphere. The ingot was remelted at least five times to achieve chemical homogeneity. Then the bulk sample with a size of φ 5 × 110 mm was produced by copper mold tilt casting technique.

The specimens for tension testing were machined into a flat dog-bone shape with gage dimensions of 0.9 mm (thickness) × 1 mm (width) × 2 mm (gage length) according to the ASTM standard E8M [30]. The room-temperature quasistatic tension test was conducted on an Instron 5582 equipment with a nominal strain rate of 4 × 10 ^−4^ s ^−1^. At least three samples were tested to guarantee the repeatability. The as-cast specimens for microstructure observations were ground, polished and then etched with a solution of 1 mL HF and 20 mL HNO_3_ onto a mirror-like surface for field electron scanning electron microscopy (FE-SEM; HITACHI SU8230, Tokyo, Japan). The hardness (H) and reduced elastic modulus (E_r_) of the specimens were determined using nanoindentation on Agilent G200 instrument with Berkovich diamond indenter (Agilent, Santa Clara, CA, USA). H and E_r_ were calculated using the Oliver–Pharr method [31]. The depth-controlled mode was employed, with an indentation depth of 200 nm and a strain rate of 0.05 s^−1^. A minimum of ten distinct regions were examined for each phase. Indentation was performed in the central region of each phase to minimize the influence from the surrounding phases and the spacing between each indentation was greater than 5 µm. The topography of the samples after tension was also examined using FE-SEM (HITACHI SU8230, Japan) and the accelerating voltage was set to 5 kV.

Phase identification for both as-cast and as-deformed specimens was analyzed using an X-ray diffractometer (Bruker D8, Bremen, Germany) from 20° to 90° with a scanning speed of 5°/min. The operating voltage was 40 kV and the instrument current was 30 mA. The crystalline phases were analyzed using the Sieve+ 2022 software. This was based on the PDF-4+ database provided by the International Center for Diffraction Data (ICDD) [32].

Transmission electron microscopy (TEM, FEI Talos F200X, FEI Tecnai F20, Waltham, MA, USA) was undertaken to further analyze microstructures in this study. Thin foils for TEM from as-cast and as-fractured samples were fabricated by ion milling (Gatan PIPS 691, Pleasanton, CA, USA), employing liquid nitrogen cooling to hinder crystallization until perforation.

## 3. Results

### 3.1. Microstructure of As-Cast Ti-BMGMCs

**Figure 1:**
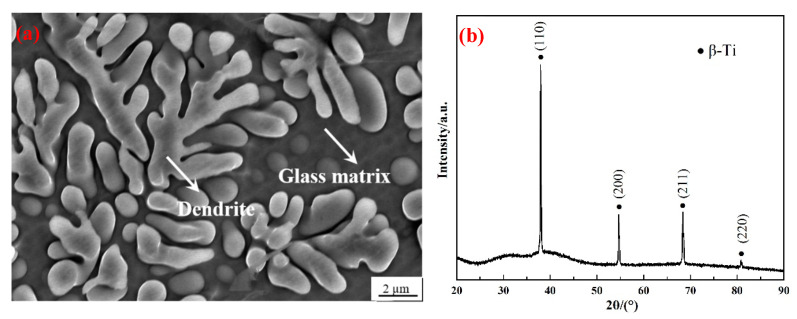
(**a**) Back scattered SEM image showing dendritic reinforcements were uniformly distributed in the glass matrix; (**b**) XRD pattern of the as-cast Ti-based BMGMCs showing crystalline peaks were overlapped with amorphous hump.

Figure 1a exhibits the back scattered SEM image of the present Ti-based BMGMCs, where dendrites were homogeneously distributed in the glassy matrix. The dendritic volume fraction, the dendritic span and the average secondary arm size were measured to be 55 ± 2%, ~30 μm and ~5 μm, respectively. As measured by SEM-attached EDS, the composition of composites is summarized in Table 1. It should be noted that the absence of Be detection is attributed to its low atomic number. As reported, almost all Be was dissolved in the amorphous matrix, contributing to its glass forming ability, and no Be was contained in the dendrite [33]. The corresponding XRD pattern is presented in Figure 1b. The diffuse hump of the amorphous phase is overlaid with diffraction peaks from a body-centered cubic (BCC) phase. This indicates the existence of a β-Ti phase in the amorphous matrix and no other crystalline phases were detected.

**Figure 2:**
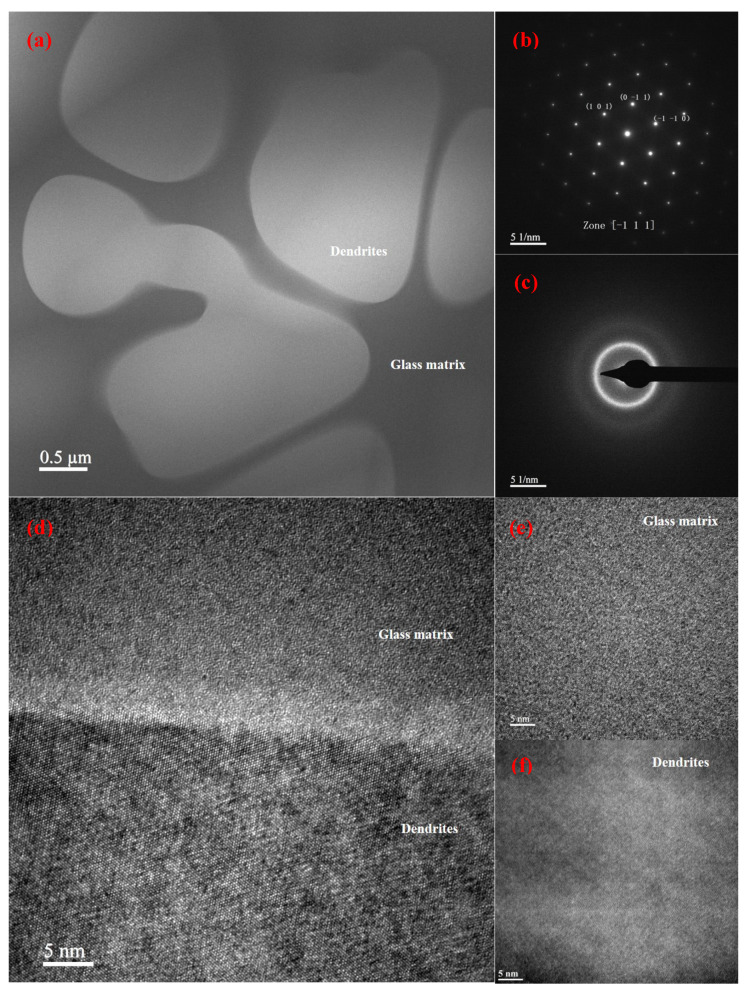
TEM and HRTEM images of the as-cast Ti-BMGMCs: (**a**) bright field TEM image, selected area electron diffraction (SAED) pattern of (**b**) β-Ti dendrites, (**c**) glass matrix, HRTEM images of (**d**) the interface between β-Ti dendritic phase and glassy phase, (**e**) β-Ti dendrites and (**f**) glass matrix.

The TEM images of the as-cast Ti-based BMGMCs are displayed in Figure 2 to reveal the microstructural features. As exhibited in Figure 2a, crystals are embedded in a featureless glass matrix. The corresponding selected area electron diffraction (SAED) patterns of the dendrites and glassy matrix are illustrated in Figure 2b,c, respectively. The SAED pattern (Figure 2b) obtained from the β-Ti phase along the zone axis of [−111] clearly exhibits distinct diffraction spots originating from a body-centered cubic (BCC) crystal structure of β-Ti. The SAED pattern (Figure 2c) acquired from the glassy matrix displays a clear diffuse ring. And there is no other diffraction spots detected. Therefore, this further confirmed the formation of BCC β-Ti and amorphous phase. The lattice parameter of β-Ti in the present BMGMCs is calculated to be a = 3.342 Å. The HRTEM image in Figure 2d shows the interface between the dendritic phase and the amorphous phase is atomically sharp. The HRTEM image of the glassy phase in Figure 2e displays a maze-like structure and that of the crystalline phase in Figure 2f further confirms no other crystalline structure was formed in the current as-cast Ti-BMGMCs.

### 3.2. Mechanical Properties

**Figure 3:**
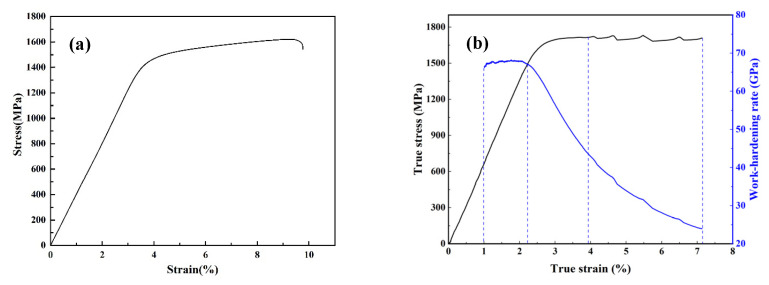
(**a**) Room-temperature compressive stress–strain curve; and (**b**) room-temperature tensile stress–strain curve and its corresponding work-hardening rate–strain curve of the Ti-based amorphous matrix composites.

Room-temperature compressive and tensile stress–strain curves are shown in Figure 3a,b, respectively, of which, the yields, fractures and corresponding elongations are summarized in Table 2. Under quasi-static compressive loading, the composites yield at ~1531 MPa, as displayed in Figure 3a. With further deformation, they were strain hardened gradually and there was no serration until the fracture failure occurred with a fracture strength of ~1684 MPa, and a fracture strain of ~9.6%.

Figure 3b plots a typical tension stress versus strain curve of the Ti-based BMGMCs. The yielding strength of the composites is ~1495 MPa with an elastic strain of 2.2%. The composites plastically deform until they reached a strength of ~1710 MPa at a strain of ~3.9%, accompanying strain hardening. They further deform in a serrated manner beyond, which is a typical characterization of bulk metallic glass. The peak serration stresses fluctuate between ~1720 MPa and ~1730 MPa. The BMGMCs fracture at a tensile strength of ~1710 MPa and at a tensile strain of ~7.1%. The work-hardening rate (dσ/dε) curve is also shown in Figure 3b. This also may give insights into the plastic flow behaviour of the given materials. During tension at room temperature, the strain hardening rate keeps constant at the beginning until the true strain is ~2.2% (yielding point) and the strain hardening rate is ~67 GPa. With a further increase in the true strain, the strain hardening rate decreases rapidly at a nearly constant decreasing rate. When true strain reaches ~3.9%, the strain hardening rate declines slowly till ~24 GPa at a strain of ~7.1%.

As summarized in Table 2, the Ti-based BMGMCs exhibit compressive and tensile strengths of approximately 1531 MPa and 1495 MPa, respectively. Moreover, they fracture at around 1684 MPa with a compression strain of approximately 9.6% and at about 1710 MPa with a tension strain of approximately 7.1%. Notably, the compressive yielding strength and strain of the BMGMCs are slightly superior to those in tension. It is common for BMGMCs to display dissimilar properties in compression and tension [34]. The uniaxial tensile loading is for unconfined deformation, whereas the uniaxial compressive loading will confine shear bands and facilitate the occurrence of multiple shear banding [12]. Therefore, the induction of shear band multiplication is easier under tension compared with compression. However, the effective inhibition of shear band propagation is less achievable under tension than compression. In consequence, the compression performance of the Ti-based BMGMCs was slightly superior to their tensile performance.

### 3.3. Fractography of Deformed Ti-Based BMGMCs

**Figure 4:**
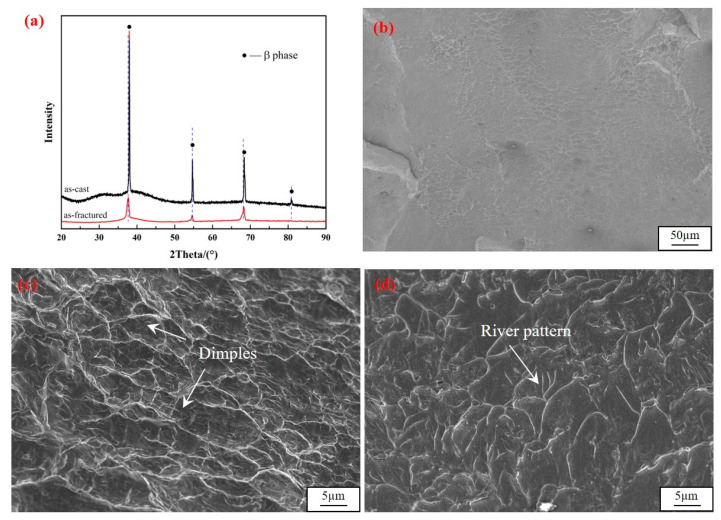
(**a**) XRD pattern of the Ti-based BMGMCs before and after tension deformation; SEM images of (**b**) fracture surface of the specimen after tension in low magnification, (**c**) fracture surface morphology of dimples and (**d**) fracture surface morphology of river-like pattern in high magnification.

To further understand the deformation mechanism of the present Ti-based BMGMCs, the XRD patterns of as-cast and as-fractured specimens are compared (Figure 4a), and also fractographs are shown in Figure 4b–d. After tension deformation, only BCC β-Ti crystalline peaks are superimposed on the amorphous humps and there are no other crystalline diffraction peaks, as displayed in Figure 4a. It confirmed that no phase transformation occurred during deformation. No difference between the XRD diffraction pattern of the Ti-based BMGMCs before and after deformation was noted. Dimples of different sizes can be observed over the fracture surface, as shown in Figure 4b,c. This is typical of a ductile fracture mechanism. In addition, a magnified image reveals a river-like pattern presented in Figure 4d, a typical feature of brittle fracture mechanisms. Therefore, this further revealed mixed fracture mechanisms under quasi-static tension loading.

**Figure 5:**
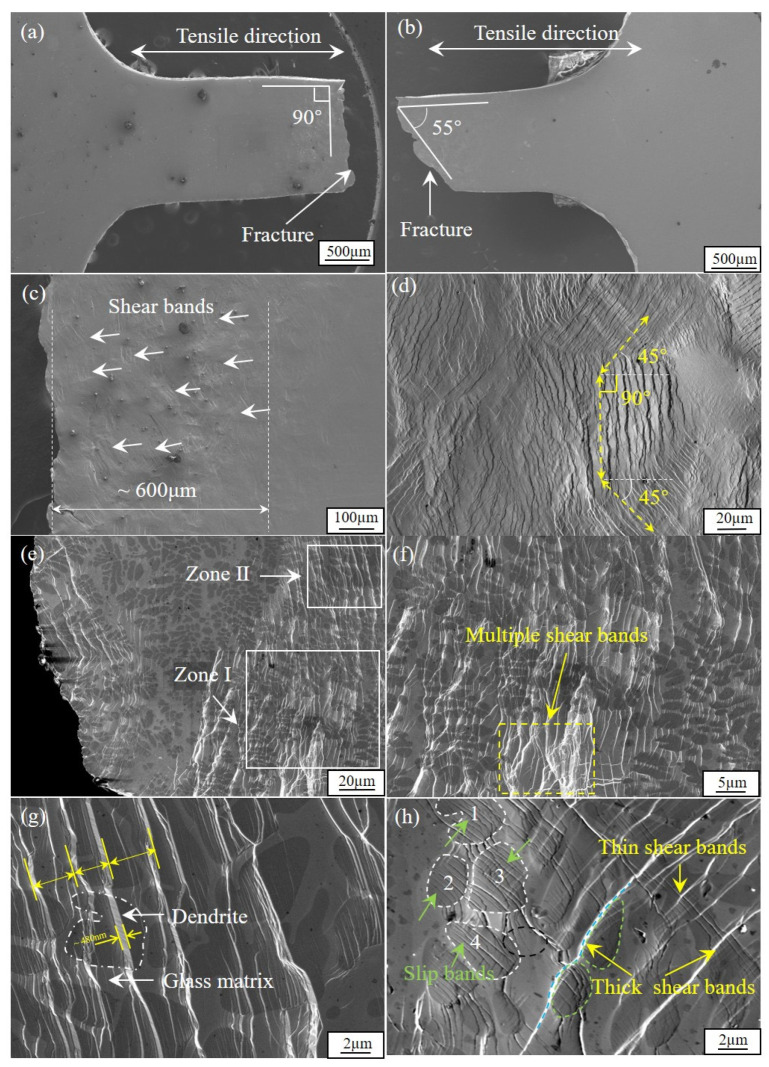
SEM images of tensile deformed Ti-based BMGMCs at room temperature: (**a**,**b**) lateral side images in low magnification; (**c**) profuse shear band near fracture; (**d**) detailed shear banding in high magnification; (**e**) deformation feature in dual phase region; (**f**) magnified image of Zone I showing multiple shear bands; (**g**) magnified image of Zone II showing shear steps; (**h**) interactions of shear bands and slip bands due to severe plastic deformation.

The image of the fractured specimen in low magnification is presented in Figure 5a,b. The width of the gauge changes slightly, suggesting a consistent elongation throughout the entire specimen without obvious necking under tensile loading. In addition, as exhibited in Figure 5a,b, the composites fractured in two modes: one is normal to the loading direction and the other occurs along a shear angle of 55°. Profuse shear bands are observed due to severe plastic deformation, as presented in Figure 5c. They are mainly distributed within a distance of 600 μm close to the fracture surface. And the density of the shear bands decreases with the increase in distance beyond 600 μm to the fracture surface. The details of shear banding are demonstrated in a higher magnification in Figure 5d, with the primary shear bands propagating mainly in three directions. These directions are approximately ±45° and 90° relative to the loading direction, as indicated by the yellow lines. Figure 5e clearly displays the deformation characteristics of the amorphous phase and the crystalline dendritic phase close to fracture surface. Further observation of Zone I (Figure 5e) in a higher magnification, as shown in Figure 5f, depicts a network structure on account of the multiplication of shear bands. The interactions between shear bands prevent its rapid propagation, and thus lead to improved ductility. As exhibited in Figure 5g, the dendrites are in dark grey and the glassy matrix are in light grey. Close inspection of Zone II (Figure 5e) demonstrated the formation of shear steps in Figure 5g, which are formed by a row of primary coarse shear bands. The width of the shear steps is within a few microns, and its height is around 480 nm. Figure 5h clearly shows the formation and propagation of slip bands within crystalline dendrites and interfacial area. It also exhibits thin secondary shear bands that are formed between coarse primary shear bands with an angle of 90°. Tiny parallel slip bands, contributing to the plastic deformation, are formed within the crystalline particles 1–4 indicated in Figure 5h, as highlighted by white dotted lines. The slip bands permeate the entire particles, across the grain boundary, and terminate in the amorphous matrix. The propagation of the shear bands is inhibited within the dendrites, as indicated by green arrows.

On the basis of the aforementioned observation of the fractography, the tensile plasticity can be ascribed to the proliferation of the shear bands and interactions between the shear bands and the slip bands. However, how both the dendritic phase and the amorphous phase deform and interact with each other and how the micromechanisms relate to tensile deformation still remains ambiguous. Therefore, further observation and analysis are required to clarify the deformation mechanism.

## 4. Discussion

### 4.1. Tensile Deformation Structures of Ti-Based BMGMCs Based on the TEM and HRTEM Analyses

**Figure 6:**
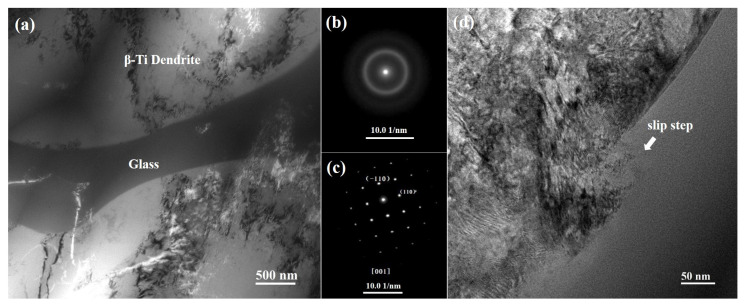
TEM micrographs in low magnification of the tension-deformed Ti-based BMGMCs: (**a**) deformed microstructures at low magnification; (**b**) SAED pattern from amorphous phase; (**c**) SAED pattern from dendritic crystalline phase; (**d**) slip step formed in the interface between dendrites and glass matrix.

To further reveal the deformation micromechanism, the deformation microstructures of the fractured samples after tension are characterized by TEM, as presented in Figure 6. Compared with the microstructure of the as-cast Ti-based BMGMCs shown in Figure 2a, the domains divided by dislocation walls in dendrites could be observed in the as-fractured samples. The SAED patterns of dendrites, shown in Figure 6c, are identified as the [0 0 1] zone axis of BCC β-Ti with a lattice parameter of a = 3.363 Å and the glassy matrix phases, shown in Figure 6b, present a diffused halo ring, further confirming an amorphous structure. The lattice parameter of the deformed alloys is a bit larger than that of the as-cast alloys. This is probably caused by the induced stress during deformation. Under low magnification, no phase transformation was detected, which is in accordance with that of the XRD result (Figure 4a). In addition, slip steps with step sizes of ~50 nm were formed in the interface between the crystalline dendrites and the glassy matrix after deformation, as presented in Figure 6d. In crystalline alloys, plastic deformation is mainly accommodated by means of dislocation movement. In contrast, shear bands are a major carrier of plasticity in an amorphous phase. And thus, the deformation behaviour is manipulated by the cooperations between the crystalline phases and the glassy phases in the BMGMCs. The formation of slip steps at the boundaries is the result of a mismatch of the abilities of the strain accommodation between the two phases during deformation. High resolution TEM images were taken to further reveal the underlying mechanisms.

#### 4.1.1. Local Amorphization

**Figure 7:**
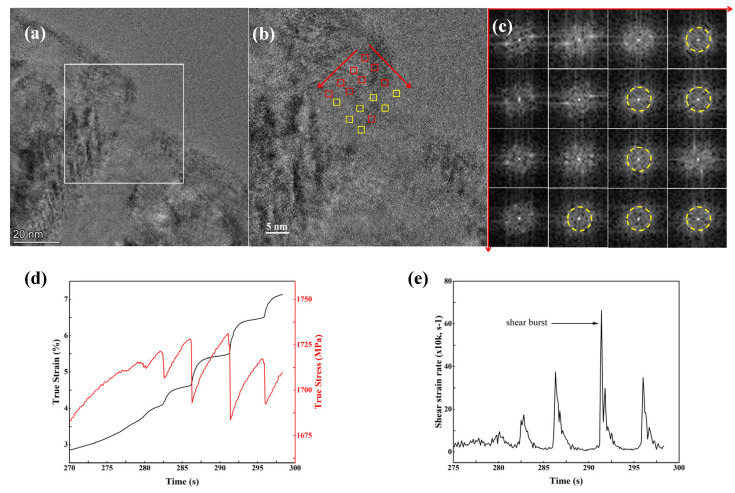
(**a**) HRTEM images showing slip step formed in the interface between the amorphous phase and dendritic phase; (**b**) enlarged area from the white square in (**a**,**c**) FFTs of red rectangle regions in (**b**), which follow the directions indicated by the arrows; (**d**) the curves of true stress vs. time and true strain vs. time; (**e**) the plot of shear strain rate $\dot{\gamma }$ vs. time.

The microstructure of the dendritic phase near the interface between the dendrites and the glass matrix after tension deformation is illustrated in Figure 7. In Figure 7a, a clear slip step, denoted by a white square, is clearly seen in the interface between the two phases. The formation of the slip step was considered to accommodate the plastic strain caused by severe deformation. To some extent, it can be regarded as a “misfit” of the strain accommodation between the crystalline phase and the amorphous phase to release local strain accumulation. Moreover, the lattice of the dendrite phase has become distorted within the dendritic phase close to the interface along the slip step, as shown in Figure 7a. The width of the lattice distortion area is about 20 nm.

In order to further reveal microstructural characteristics after deformation along the interfacial slip stepped area within the dendrites, a square with a size of 10 by 10 nm was selected and then 16 small areas with a size of 1 by 1 nm were chosen evenly within it for fast Fourier transformation (FFT), as displayed in Figure 7b,c. Several amorphous-like regions were found, as marked by circles in the FFT pattern in Figure 7c, confirming the formation of local disordered amorphization within the dendritic crystalline phase. The local amorphization phenomenon has also been mentioned before [35,36,37]; however, no explanation has been given. The supposed local structure changes, i.e., local amorphization, results in the release of accumulated shear strains, thus preventing the propagation of shear bands in the nanostructured composites. In order to understand the formation of local amorphization in the BMGMC, the local shear strain rate was examined. The local shear strain was expressed by Tong et al. [38] as

$$\gamma =\frac{\Delta \varepsilon H}{hcos\theta }$$

where *H* is the open gauge length of the tensile sample, *h* is the thickness of the shear bands, and *θ* is the averaged shear angle. Here, *H* = 2 mm, *h* ≈ 10 nm [31,32] and *θ* = 90°/55°. Hence the local shear strain rate can be determined by analyzing the strain vs. time curve, as shown in Figure 7d.

$$\dot{\gamma =\frac{H}{hcos\theta }\times \frac{\partial \Delta \varepsilon }{\partial t}}$$

where $\dot{\gamma }\;$ is the local shear strain rate. The plot depicting the relationship between $\dot{\gamma }$ and time is illustrated in Figure 7e. As mentioned in Section 3.3, two shear angles were observed at 90° and 55°, respectively. Thus, the magnitude of the local shear strain rate is in the order of 10^3^–10^4^ s^−1^ up to infinity, which is more than seven magnitude orders higher than the quasistatic tensile strain rate and is also in accordance with that reported before [38]. The local high strain rates also correspond to those of the impact loading [39,40]. The high local strain rates imply the occurrence of local amorphization at the interface between dendrites and the glassy matrix.

The occurrence of serrations in the tensile stress–strain response is a notable feature for the BMGMCs, as shown in Figure 3b. Several studies on the mechanical deformation of Ti-based BMGMCs have reported a similar serrated flow feature [9,23]. In their studies, serrations are correlated to the size of the dendrite. Coarse dendrites with sizes larger than 10 μm could prevent stress localization effectively and thus exhibit less or no serrations. However, in our study, the size of the dendrites range from 1 to 5 μm, much less than 10 μm, and they cannot resist stress localization. Therefore, stress localization induces local amorphization, accounting for the occurrence of serrations in the stress flow to some extent in our study.

#### 4.1.2. BCC → HCP Structural Transition

**Figure 8:**
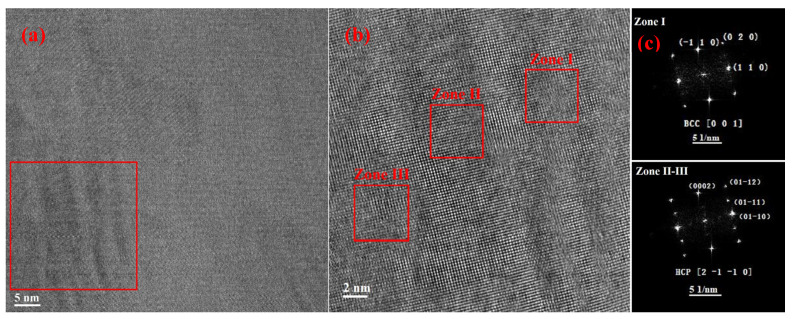
(**a**) HRTEM image of tension-fractured Ti-based BMGMCs showing microstructural characteristics in the crystalline region close to slip step; (**b**) enlarged image of the red square in (**a**), which shows atomic microstructure of the dendrite phase close to slip step; (**c**) FFTs of Zone I, Zone II and Zone III indicated by red squares in (**b**), respectively.

Figure 8a is an HRTEM image of the Ti-based BMGMCs, showing crystalline microstructural change after tension deformation close to the slip step. The local enlarged image and associated SAED patterns of the transition region, presented in Figure 8b,c, demonstrate a distinct orientation relationship (OR) between the BCC and HCP structures, i.e., [0 0 1] BCC//[2 −1 −1 0] HCP, (−1 1 0) BCC//(0 0 0 2) HCP. This demonstrates that a local phase transformation from a BCC to an HCP structure occurred during tensile deformation. The spot of (0 1 1) directly convinces the presence of α phase, rather than ω phase. The size of the phase-transformed areas is in several nanometer scales. Comparing the identified crystalline phases in the dendrites before and after the tensile fracture, it is suggested that that increased localized stress contributes to the formation of nano-sized α phase locally.

As shown in Figure 8b,c, (1 1 0) BCC-β is parallel to (0 1 −1 0) HCP-α, with the former in a d-space of 2.263 Å and the later in a d-space of 2.323 Å; (−1 1 0) BCC-β (2.327 Å) is parallel to (0 0 0 2) HCP-α (2.326 Å), both in similar d-spacings; and the zone axis of [0 0 1] BCC-β is parallel to zone axis of [2 −1 −1 0] HCP-α [41]. Therefore, stress-induced phase transformation will lead to a lattice expansion, resulting in stress redistribution. Upon loading, lattice expansion may introduce extra stress to the adjacent glassy matrix due to the tight bonding between dendrites and matrix [12,14]. The interfacial region in the glass will be more strained, acting as nucleation sites for shear banding. Considering the confinement of the dendrites by the amorphous phase, the stress within it cannot be fully released as in the free crystals. As a result, “spots”, where α phases (or HCP structures) are located, form and shear banding nucleation sites produce.

**Figure 9:**
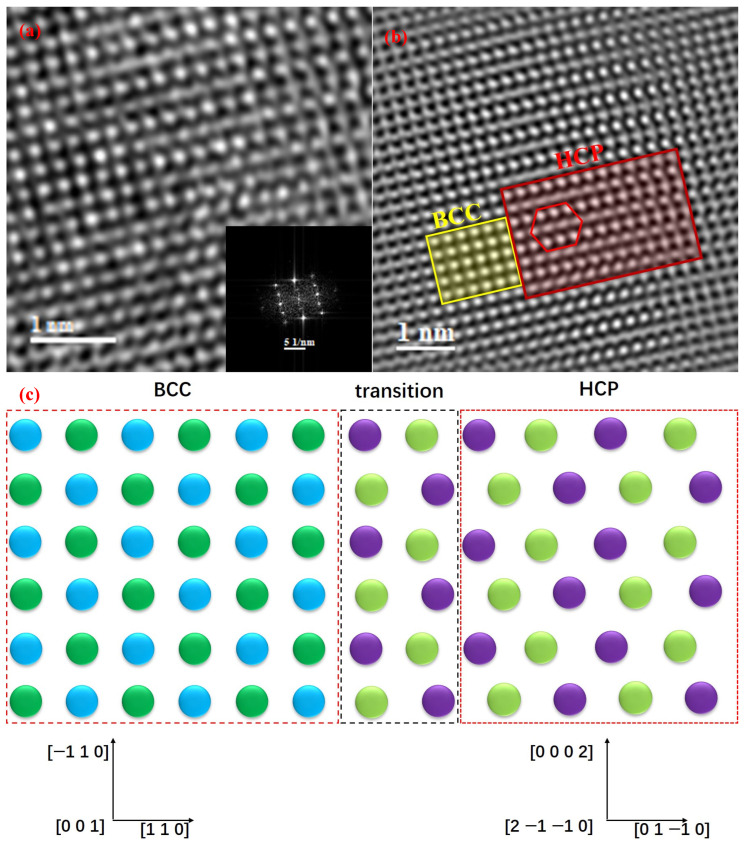
(**a**) An HRTEM image of the region showing a BCC to HCP transition and inset shows a corresponding FFT pattern; (**b**) IFFT of (**a**) shows a clear transition from BCC to HCP; (**c**) a schematic diagram of the transition from BCC to HCP.

Figure 9a presents an HRTEM image capturing the region of a structural transition from BCC to HCP with an orientation following [0 0 1] BCC//[2 −1 −1 0] HCP and (1 1 0) BCC//(0 1 −1 0) HCP. The corresponding IFFT in Figure 9b clearly depicts the lattice structural change from the BCC to HCP transition, where the BCC structure is denoted in an orange square and the HCP structure is in a red square. As *in situ* observation [42], the phase transformation is proposed to be completed in the non-diffusive way, i.e., through a dislocation-mediated process. In the BCC phase, the d-spacing of plane (1 1 0) along [1 1 0] is 2.263 Å. In the transformed HCP phase, the d-spacing of plane (0 1 −1 0) along (0 1 −1 0) is 2.323 Å. The transition from BCC to HCP introduces lattice expansion through a decrease in the inclined angle of the (−1 1 0) plane to [1 1 0] in BCC, and it is proposed that the expansion is accomplished by partial dislocation dipoles [43,44]. In addition, the atomic shuffling also contributes to the slight lattice expansion of 2.65%. The synergistic cooperation of dislocation gliding and atomic shuffling accomplishes the transition from a BCC to an HCP structure, as schematically demonstrated in Figure 9c. In consequence, a structural transition could potentially contribute to the alleviation and redistribution of the concentrated stress and improve the global plasticity.

### 4.2. Deformation Mechanism during Tension

**Figure 10:**
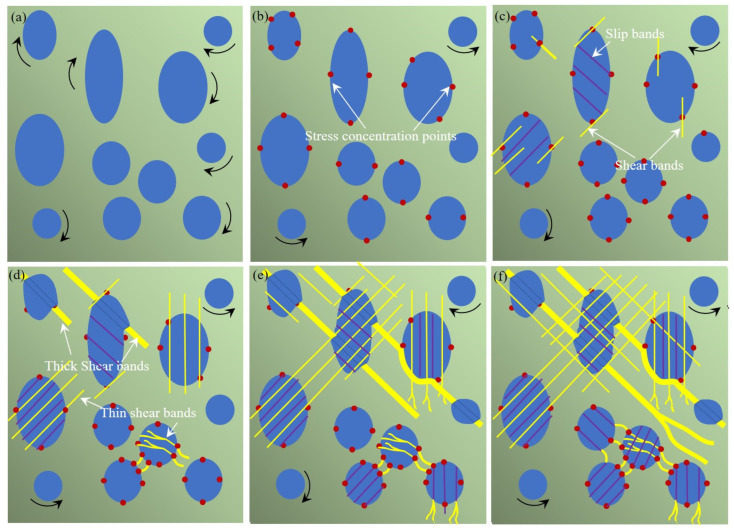
Schematic illustrations of the microstructural evolution for Ti-based BMGMCs during different tension deformation stages: (**a**,**b**) elastic stage, during which dendrites deformed initially, leading to interfacial stress concentration; (**c**–**f**) plastic stage, both dendrites and glassy matrix deformed, shear bands formed and propagated, leading to the final failure.

The deformation behaviour can be understood in the following way and the mechanisms of the plastic deformation of the Ti_42.3_Zr_28_Cu_8.3_Nb_4.7_Ni_1.7_Be_15_ BMGMCs has been schematically demonstrated in Figure 10a–f. In the initial stage of deformation, both the crystalline phase and the amorphous matrix phase are elastically deformed. According to the nanoindentation results, crystalline dendrites are comparably softer than the glassy matrix with a modulus value of 92.3 GPa vs. 103.3 GPa and a hardness value of 5.7 GPa vs. 7.3 GPa. Therefore, the yielding of the β-Ti phase in the current composite is primarily initiated by nucleation and dislocation gliding, resulting in a higher stress-bearing capacity of the amorphous phase due to the interfacial stress concentration.

As deformation continues, the high stress concentration at the interface triggers the activation of shear transformation zones (STZs) in the amorphous phase. The percolation of STZs will further lead to the formation of shear bands [45] and thus the amorphous matrix will be plastically deformed. The presence of the shear bands within the matrix indicates the macroscopic yielding of the BMGMCs. Following yielding, the shear bands are predominantly confined to the intercrystalline amorphous phases, as shown in Figure 10c. Upon further deformation, interfacial stress continues to accumulate and may be released by extending shear bands to both sides of the interface. The extension of shear bands into the dendrites results in the formation of slip steps, accompanying a high local strain rate, as presented in Figure 10c,d. The high local strain rate leads to the localized amorphization close to interfacial region, causing the serrations on the stress–strain curves. On the other hand, the shear bands may expand into the amorphous matrix and then reach the other dendrites across the glassy matrix. Then, other local amorphization events will be activated in the further deformation. In addition, accumulating concentrated stress causes the localized structural transition from BCC to HCP in nanoscale. The localized structural transition benefits stress relaxation and redistribution, thus improving deformation performance.

## 5. Conclusions

In conclusion, the present study investigated a novel plastic deformation mechanism of Ti-based BMGMCs containing meta-stable β-Ti dendrites under tension. The plastic deformation of the Ti-based BMGMCs after yielding is mediated by a synergistic action, which consists of shear banding in a glassy matrix, localized amorphization and a BCC to HCP structural transition in the β-Ti dendrite. The formation of local disordered amorphization within the dendritic crystalline phase occurs with a width of ~20 nm. The high local strain rate during the deformation process can reach values up to 10^3^ ~ 10^4^ s^−1^, potentially approaching infinity, thereby further substantiating the potential occurrence of localized amorphization. The formation of an HCP structure is induced by localized stresses and has been confirmed to be α phase rather than ω phase. The structural transition from β to α results in lattice expansion, leading to stress alleviation and redistribution. The occurrence of localized amorphization and the transition from the BCC to HCP structure are attributed to a high local strain rate and concentrated interfacial stresses, leading to the manifestation of serrations and extended global plasticity. The finding expands the comprehension of the deformation mechanism of the Ti-based BMGMCs and also sheds light on materials design and performance improvement.

## Figures and Tables

**Table 1:** Elemental contents in the as-cast Ti-based BMGMCs (at%), dendrite volume fraction (V_d_), dendritic span (λ_d_) and average secondary dendritic arm size (S_d_).

	Zone	Composition (at%)						V_d_ (%)	λ_d_ (μm)	S_d_ (μm)
		Ti	Zr	Cu	Ni	Nb	Be			
Ti-BMGMCs	Composite	42.3 ± 0.03	28 ± 0.1	8.3 ± 0.1	1.7 ± 0	4.7 ± 0.02	15 ± 0.1	55 ± 2	~30	~5
	Dendrites	53 ± 1.3	32.9 ± 0.2	6.1 ± 1.0	1.4 ± 0.3	6.6 ± 0.2	-			
	Matrix	39.1 ± 0.4	40.8 ± 0.4	13.3 ± 0.1	3.4 ± 0.04	3.4 ± 0.1	-			

**Table 2:** Room-temperature mechanical properties of the Ti-based bulk metallic glass matrix composites.

	Yielding Strength (MPa)	Ultimate Strength (MPa)	Elongation (%)
Compression	1531	1684	9.6
Tension	1495	1710	7.1

## Data Availability

The original contributions presented in the study are included in the article, further inquiries can be directed to the corresponding author.
